# Trends in reasons for emergency calls during the COVID-19 crisis in the department of Gironde, France using artificial neural network for natural language classification

**DOI:** 10.1186/s13049-021-00862-w

**Published:** 2021-03-31

**Authors:** Cédric Gil-Jardiné, Gabrielle Chenais, Catherine Pradeau, Eric Tentillier, Philippe Revel, Xavier Combes, Michel Galinski, Eric Tellier, Emmanuel Lagarde

**Affiliations:** 1 Inserm, ISPED, University of Bordeaux, Bordeaux Population Health Research Center Inserm U1219 Injury Epidemiology Transport Occupation team, Bordeaux, France; 2 University Hospital of Bordeaux, Pole of Emergency Medicine, Bordeaux, France

**Keywords:** Emergency medical communication centers, COVID-19, Lockdown, Public health

## Abstract

**Objectives:**

During periods such as the COVID-19 crisis, there is a need for responsive public health surveillance indicators in order to monitor both the epidemic growth and potential public health consequences of preventative measures such as lockdown. We assessed whether the automatic classification of the content of calls to emergency medical communication centers could provide relevant and responsive indicators.

**Methods:**

We retrieved all 796,209 free-text call reports from the emergency medical communication center of the Gironde department, France, between 2018 and 2020. We trained a natural language processing neural network model with a mixed unsupervised/supervised method to classify all reasons for calls in 2020. Validation and parameter adjustment were performed using a sample of 39,907 manually-coded free-text reports.

**Results:**

The number of daily calls for flu-like symptoms began to increase from February 21, 2020 and reached an unprecedented level by February 28, 2020 and peaked on March 14, 2020, 3 days before lockdown. It was strongly correlated with daily emergency room admissions, with a delay of 14 days. Calls for chest pain and stress and anxiety, peaked 12 days later. Calls for malaises with loss of consciousness, non-voluntary injuries and alcohol intoxications sharply decreased, starting one month before lockdown. No noticeable trends in relation to lockdown was found for other groups of reasons including gastroenteritis and abdominal pain, stroke, suicide and self-harm, pregnancy and delivery problems.

**Discussion:**

The first wave of the COVID-19 crisis came along with increased levels of stress and anxiety but no increase in alcohol intoxication and violence. As expected, call related to road traffic crashes sharply decreased. The sharp decrease in the number of calls for malaise was more surprising.

**Conclusion:**

The content of calls to emergency medical communication centers is an efficient epidemiological surveillance data source that provides insights into the societal upheavals induced by a health crisis. The use of an automatic classification system using artificial intelligence makes it possible to free itself from the context that could influence a human coder, especially in a crisis situation. The COVID-19 crisis and/or lockdown induced deep modifications in the population health profile.

**Supplementary Information:**

The online version contains supplementary material available at 10.1186/s13049-021-00862-w.

## Introduction

Coronavirus 2019 Disease (COVID-19) caused by Severe Acute Respiratory Syndrome Coronavirus 2 (SARS-CoV-2) was detected for the first time in December 2019 in China [1]. Since then, as of first November 2020, more than 40 million cases and more than 1,100,000 deaths have been reported worldwide [2].

Health-related surveillance data are indispensable for the adjustment of public health policies aimed at curbing the spread of the virus and protecting vulnerable populations, for adapting the means of caring for the most seriously ill, and for assessing the indirect consequences of the pandemic and of the measures implemented to control it: lockdown, movement restriction, social distancing and quarantine for some countries.

Many countries have relied on an extrapolation of classic infection-control and public-health measures to contain the COVID-19 pandemic, similar to those used for Severe Acute Respiratory Syndrome virus in 2003 and H1N1 influenza virus in 2009. These range from extreme quarantine measures in China to painstaking detailed contact tracing with hundreds of contact tracers (e.g., Singapore, Hong Kong, South Korea). However, these measures may not be effective in 2020 for tackling the scale of COVID-19 [3]. Monitoring, surveillance, detection and prevention of COVID-19 have launched a new challenge on a global scale and data sciences technologies have so far failed to address it with a few notable exceptions [4].

The monitoring of the number of people who test positives by Reverse Transcriptase-Polymerase Chain reaction (PCR) or with chest computerized tomography images with characteristic lung damage is the most reliable indicator of the number of people with the virus. It is, however, heavily dependent on the screening strategy, which varies greatly from one country to another. The number of people entering an Emergency Room (ER) with symptoms suggesting SARS-CoV-2 infection was implemented nationally in France on March 16, 2020, with specific coding of the main diagnosis in ER summary reports centralized by Santé Publique France in the OSCOUR® Emergency Department Surveillance Network [5] set up in 2004. The French health authorities are also monitoring the number of COVID-19 patients hospitalized, admitted to intensive care units, and the number of deaths in hospital and in nursing homes (EHPAD).

All these tools, completed by a large set of ad-hoc surveys and specific surveillance systems have been mobilized to monitor the course of the epidemics and its consequences. For example, analysis of the registry of the Paris-Sudden Death Expertise Center observed a transient increase in cardiac arrest incidence during the COVID-19 crisis [6]. A similar observation was reported in Lombardy, Italy, a region heavily impacted by the COVID-19 pandemic [7].

Reports of call content to emergency medical communication centers (EMCC) is a source of information that needs to be considered for health surveillance during such a pandemic. By dialing 15, French citizens can get medical advice and, if necessary, a medical mobile care unit can be sent to the scene. In addition, the health authorities had instructed the population to call 15 in the case of symptoms or concerns, and to avoid spontaneous uninvited visits to the ER [8].

We used an automatic classification tool based on an artificial neural network language model we recently adapted for free-text clinical notes [9] to identify the main reasons for calls to the Gironde EMCC, in order to monitor trends in the nature of these calls before, during and after the national lockdown established in France on March 17, 2020 and eased on May, 11, 2020.

## Method

### Setting

The Gironde department (1.6 million inhabitants) is served by a medicalized EMCC known as SAMU 33 (Service d’Aide Médicale Urgente de la Gironde) which answers calls to the French toll-free number dedicated to medical emergencies (the “15”). A call is first received by a medical assistant, and then an emergency physician or a general practitioner (depending on the severity of the case) decides on the appropriate response, from medical advice to the dispatch of an ambulance or a mobile intensive care unit [5].

### Clinical reports

For all cases handled, a clinical report is created in the form of a computerized free-text note and updated by a medical assistant and a physician through the various telephone interactions with the patient, family, witnesses, and then with the paramedics if applicable. These clinical notes contain all the elements that make it possible to know the circumstances of the event at the origin of the call and the clinical observations made by the protagonists, whether they are witnesses, the patients themselves, or the medical personnel involved on site or responding to the call.

### Classification of clinical reports using the GPT-2 neural language model

Over the past 10 years, neural language models have progressively taken the largest share in the field of natural language, with applications like machine translation, document classification, text summarization and speech recognition.

New levels of performance have only recently been achieved with the use of models based on the concept of attention, which consists in learning dependencies between words in a sentence with-out regard to their distances. This mechanism has been implemented in a sequence to sequence neural network model, the Transformer architecture, proposed in 2017 [10]. One of the latest examples is the Generative Pretrained Transformer 2 (GPT-2), published in February 2019 by OpenAI. GPT-2 is a large transformer-based language model trained on a dataset of 8 million web pages [11]. Beyond the capability to generate coherent texts, Transformer models have the potential to perform other tasks such as question answering and document classification with a limited number of training examples. The training of the model is performed in two distinct phases [12]: the first pre-training unsupervised phase (i.e. not needing a human classification), consists in exploitation of a text corpus. In our application, this consisted in the 312,367 clinical reports of the year 2018. This leads to the ability of automatic text generation (see Additional file 1). The relevance of these synthetic sentences suggests that the networks learn contextual semantic representations. The second training phase, this time a supervised one (i.e. using examples classified by humans), creates a system able to perform the specific classification tasks.

The 117-million parameters version of the GPT-2 model was trained on a workstation with one Nvidia GeForce RTX Titan Graphic Processing Unit with 24GB of video random access memory. The computation time for the pre-training phase was 26 h per epoch (an epoch corresponds to the entire training set going through the entire network once). On average, 12 samples were processed per second in the training phase.

### Extraction of free-text digital calls reports from the EMCC of the Gironde department, France

We retrieved call reports from the digital medical record system of the EMCC of the Gironde department, France. Five EMCC datasets were extracted corresponding to the years 2016, 2017, 2018, 2019 and 2020. The three 2016, 2017, 2018 datasets consisted of 888,469 calls with a free-text clinical report and were used both for the unsupervised pre-training of the GPT-2 model and for the building of the training sample based, when available, on standardized diagnosis coded during the calls. The 2019 dataset corresponded to the 302,925 free-text clinical call reports of the year 2019. A random sample of this dataset was manually coded by a trained pool of emergency nurses for validation. The trained and validated models were applied to the 2020 dataset which corresponded to the COVID-19 period, with 255,556 handled calls with a free-text report from January 1, 2020 to September 30, 2020. This provided a classification of reasons for calls before (January 1–March 16), during (March 17–May 10) and after (May 11–September 30) COVID-19 lockdown.

### Selected groups of reasons for calls

A first model was built by grouping calls according to the main reasons into 12 broad categories (chest pain, gastroenteritis and abdominal pain, flu-like symptoms and breathing difficulties, focal neurologic deficit and stroke, road traffic crash, violence, suicide and self-harm, injury other than violence, self-harm and road crash, pregnancy and delivery problems, malaise with loss of consciousness, stress and anxiety). Many symptoms or situations did not fall into these broad categories and were grouped into a thirteenth “other” category.

Another distinct model was built to count calls for which an acute alcohol intoxication was involved.

### Classification procedure

Classification models were built and validated using a five-phase procedure:

(i) A total of 888,469 records with call reports of 2016, 2017 and 2018 were used to pre-train the GPT-2 model in an unsupervised manner with 1 epoch.
(ii) A dataset of training examples for supervised training was built extracting the 690,103 (out of 888,469) call reports from the same 2016, 2017, 2018 years with a diagnosis code manually coded in real time by the people handling calls (medical regulation assistants, emergency physicians and general practitioners); The pre-trained model was then fine-tuned using these training labelled examples to build the model used to classified main reasons for call in 12 categories and a distinct model to identify calls for which an acute alcohol intoxication was involved.
(iii) These two models were validated using the manual coding of a 39,907 random sample from the 2019 dataset.
(iv) All 254,633 call reports of 2020 (January to September) were classified using the two models built in the previous steps;

### Emergency room admissions with suspected COVID-19, SARS-CoV2–2 PCR tests result and general practitioner house call network (SOS Médecins)

We retrieved daily aggregated data of ER admission with suspected COVID-19, the daily number of SARS-Cov-2 PCR positive tests in the department of Gironde, and the daily number of calls to the general practitioner house call network (SOS Médecins) [13] from the national public health agency “Santé Publique France”. All data are available on the Geodes website (https://geodes.santepubliquefrance.fr/).

### Analysis

For all call reasons, a probability cutpoint was determined in order to optimize the accuracy of the number of calls estimated by the model. This was done by selecting the cutpoint for which precision and recall are equal in the validation sample (i.e. when the number of false positive predictions equals the number of false negative predictions). The confidence of the method was assessed using a bootstrap procedure with 10,000 1:1 random partitions of the validation sample. For each partition (and each reasons), the cutpoint is selected using the first half of this partition sample and is the one for which precision equals recall. The difference between the manually determined prevalence of the given reasons and the predicted prevalence (using the selected cutpoint) is then computed in the second half of the sample. Median bias and 95% confidence interval were derived from the distribution of these 10,000 bias measures. Estimated daily counts of calls to EMCC were plotted against time with a 7-days moving average window to smooth the one-week periodicity in calls, which were systematically 25% more frequent on Saturday and Sunday.

## Results

### Model training, parametrization and validation

The unsupervised training phase led to a model capable of generating artificial texts with the same structure as the learning texts, containing essentially the same clinical notions, but whose coherence is often inconsistent (see examples in the Additional file 1). The content of these synthetic reports however suggests that the model has learned contextual semantic representations.

The training sample included from 1829 (road traffic crash) to 412,218 (other reasons) samples per group (see Table 1). Performance statistics computed using the 39,907 manually-coded samples are shown in Table 1. Area under the ROC curve ranged from 0.785 to 0.990.

**Table 1:** Training sample size (from 2016 to 8 datasets) and validation from manually coded samples from 2019 dataset

Reasons	Training sample size	Max F1	AUC	Accuracy	Proportion in 2020	95% confidence interval using bias estimated by bootstrap^a^
Main reasons for EMS calls						
Chest pain	29,310	0.800	0.987	0.978	0.057	0.0542–0.0598
Gastroenteritis and abdominal pain	63,446	0.710	0.958	0.946	0.082	0.0777–0.0863
Flu-like symptoms and breathing difficulties	72,323	0.683	0.958	0.929	0.151	0.1461–0.1559
Focal neurologic deficit, stroke	5951	0.698	0.978	0.991	0.0135	0.0118–0.0152
Road traffic crash (RTC)	1829	0.799	0.980	0.988	0.0233	0.0214–0.0252
Violence	3158	0.636	0.984	0.991	0.011	0.0092–0.0128
Suicide and self-harm	5904	0.654	0.969	0.988	0.015	0.0131–0.0169
Injury other than violence, self-harm and RTC	120,007	0.694	0.938	0.887	0.166	0.1596–0.1724
Pregnancy and delivery problems	6222	0.804	0.990	0.994	0.013	0.0116–0.0144
Malaise with loss of consciousness	41,468	0.492	0.935	0.958	0.035	0.0313–0.0387
Stress and anxiety	12,198	0.479	0.877	0.956	0.046	0.042–0.05
Other reasons	412,218	0.673	0.785	0.746	0.385	0.3754–0.3946
Alcohol intoxication	8934	0.712	0.982	0.979	0.033	0.0303–0.0357

^a^ As estimated by bootstrapping (*N* = 10,000) the validation sample

The optimal cutpoints to minimize bias in prevalence estimates were chosen using the same manually-coded sample of 39,907 call reports. Bootstrap 95% confidence interval assessment showed that the method provided narrow confidence intervals (Table 1).

### Reasons for calls during the Covid-19 crisis

In 2020, the median daily number of calls to the EMCC with a report was 892 with a total number of 254,633 and a peak of 1926 on March 14. The distribution of the estimated number of calls per group of reasons and according to the lockdown period is shown in Table 2.

**Table 2:** Reasons for calls to EMS in 2020 as determined by the GPT-2 model. Calls per day and per motive

Motives	Before lockdown (calls per day)	During lockdown (calls per day))	After lockdown (calls per day)
Total	*N* = 79,672	*N* = 49,743	*N* = 125,218
Main reasons for EMS calls			
Chest pain	5.1	7.2	5.5
Gastroenteritis and abdominal pain	8.9	7.3	8.2
Flu-like symptoms and breathing difficulties	20.7	22.7	8.7
Focal neurologic deficit, stroke	1.3	1.3	1.4
Road traffic crash (RTC)	2.2	0.9	3.0
Violence	0.10	0.08	0.13
Suicide and self-harm	1.3	1.4	1.6
Injury other than violence, self-harm and RTC	15.0	11.4	19.7
Pregnancy and delivery problems	1.2	1.3	1.4
Malaise with loss of consciousness	3.4	2.1	4.0
Stress and anxiety	3.8	5.5	4.8
Other reasons	35.4	36.6	41.3
Alcohol intoxication	3.0	2.3	3.9

**Fig. 1:**
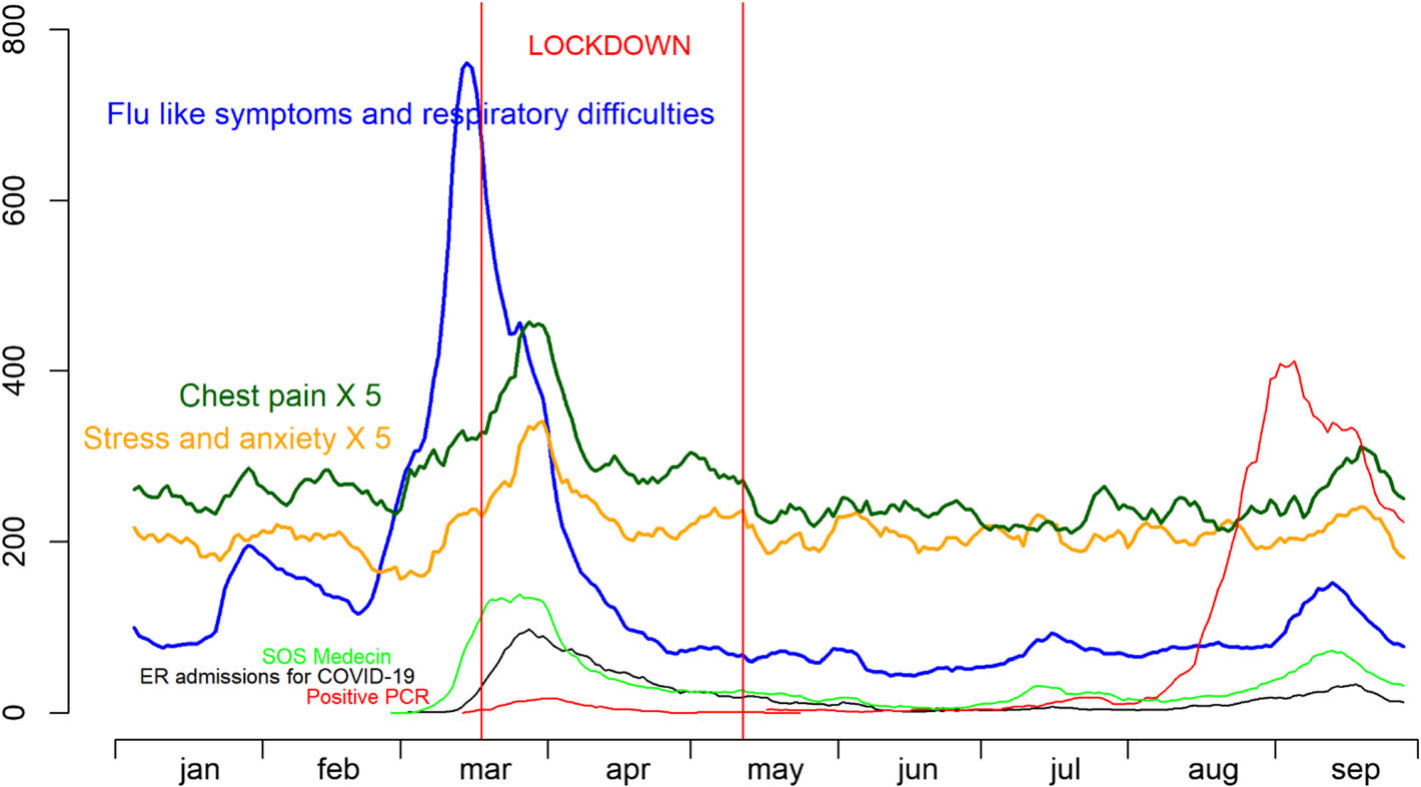
Number of calls to the Gironde to emergency medical communication center in 2020 by reasons from GPT-2 classification with a significant increase around the onset of the lockdown period

Three groups exhibited a peak at the onset of the lockdown period. Figure 1 shows that calls for flu-like symptoms and breathing difficulties began to rise on February 21, 20 days before the rise in ER admission for COVID-19, and peaked on March 14 with 928 calls out of a total of 1926 calls that day. By the 16th day after February 21, the number of calls in this group had reached 403 calls per day, which is higher than all levels over the past 5 years. Starting from March, 1, 2020, the correlation between daily calls for flu-like symptoms and daily ER admissions with a delay of 14 days was 0.80 (*p* < 10^− 15^; Pearson’s test). Two other reasons, chest pain and stress and anxiety, were found to peak 12 days after March 14, with a curve of the same shape as the number of ER visits for COVID-19.

**Fig. 2:**
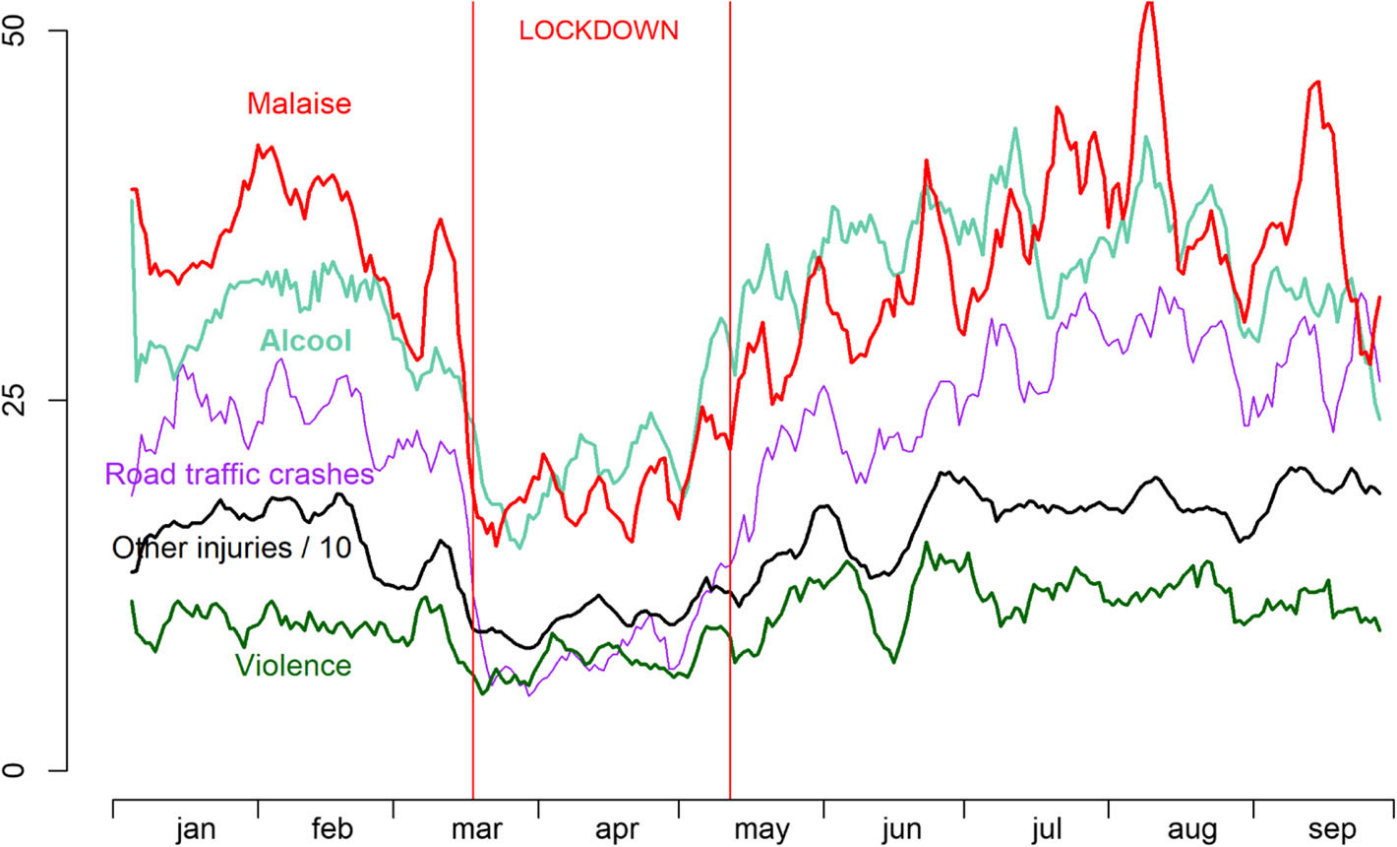
Number of calls to the Gironde to emergency medical communication center in 2020 by reasons for calls from GPT-2 classification with a significant decline around the onset of the lockdown period

Figure 2 shows that calls for alcohol intoxication, road traffic crash and malaise with loss of consciousness experienced a sharp decline around the onset of lockdown and that these trends started several days before the first day of lockdown. In a lesser extent, the latter curves were parallel with those for violence and injuries other than violence and road crash. Finally, no noticeable trends in relation to lockdown was found for other groups of reasons including gastroenteritis and abdominal pain, stroke, suicide and self-harm, pregnancy and delivery problems.

## Discussion

The number of calls to EMCC showed a significant spike centered on the March 14, the day before the closure of all non-essential public places in France, three days before lockdown. The main reasons were related to flu-like symptoms (cough and/or fever), followed 14 days later by a peak in ER admissions, in calls for chest pain and calls for stress and anxiety. Calls for road traffic crashes, malaises with loss of consciousness, non-voluntary injuries and alcohol intoxication fell by 59, 38, 24 and 23% respectively during lockdown.

Probably the most interesting finding of our study is the delay we observed between the rise in calls for flu-like symptoms (mainly cough and fever) and the rise in ER visits for suspected COVID-19. Thus, the curve began to rise 20 days before the increase in ER visits. By the 16th day after the start of this rise, the levels reached a level higher than any levels in the past 5 years. One could hypothesize that the peak of calls recorded around March 14 was due by the concern, if not anxiety, caused by the announcement on television of the closure of public places by the French President on that day. However, in a more affected part of the country, the Ile-de-France region, the peak was reached much earlier (10 days earlier) [13], suggesting that most of the calls we recorded were more motivated by symptoms than by concerns raised by communication by the authorities. Further, a spike in calls for stress and anxiety was measure 14 days later. EMCC call content is therefore probably the most predictive early indicator of the start of the epidemic, as recently shown by Riou and colleagues who found in the Ile-de-France region a strong correlation between calls regarding suspected COVID-19 and the number of patients in intensive care, with a delay of 23 days [14]. This is why this is considered for the monitoring of a potential relapses in the epidemic [15]. Finally, while the number of calls for flu-like symptoms proved to be an early and relevant signal, its intensity was probably increased by the request of the authorities not to go directly to the ER and to contact instead the EMCC.

An important difference between the work of Riou and colleagues and this study is that our process was clearly agnostic to the COVID-19 epidemic or to lockdown, as the automatic classification used models trained using reports from previous years. This results in a procedure that remains independent of the COVID-19 epidemic and lockdown, which would have influenced human codification. The signal thus obtained depends less on the context and is more likely to be an indicator of the actual public health situation. More generally, the added value of using an automatic classification procedure based on a natural language processing model is that it frees us from the context in which the reported events are coded. For this reason, we did not use the coded diagnoses at the time of the call to observe trends. In addition, these diagnoses were absent from one-third of the reports.

In the context of the COVID-19 epidemic, several research teams have used a similar approach, attempting to investigate the internet or social media to build early indicators of the epidemic [16, 17]. However, no such signal could be found from a Google keyword search [18], as the peak for cough, fever, coronavirus or COVID-19 was not reached until the week of 15–21 March.

Contrary to what was observed in Paris [19], no increase in calls related to cardiac arrest was observed in our study. This observation supports the hypothesis that the transient twofold increase in out-of-hospital cardiac arrests observed in Paris and its suburbs could be due to COVID-19 infections and to pandemic-related health system issues in heavily impacted regions. This was clearly not the case in the Gironde department where EMCC and intensive care units have never been overwhelmed.

A very significant decrease in calls for malaise with loss of consciousness, and to a lesser extent for strokes, was observed, starting one week before lockdown. This paralleled the sudden drop in ER visits that was observed in many countries that issued a statewide stay-at-home order [20], raising concerns that patients who needed medical care were not presenting to the hospitals and, for example, that stroke patients arrived too late to receive tissue plasminogen activator. The actual overall public health impact of this phenomenon will have to be carefully assessed when we have enough hindsight to appreciate its medium-term health consequences.

The decrease in calls associated with interpersonal violence and alcohol intoxication is less surprising as it is probably due to the reduction in social interactions. Interestingly, the figures returned to normal levels by the end of the lockdown period. Early on, concerns were raised about the risk of domestic violence as a result of lockdown [21]. This was not confirmed here by calls to EMCC. Although, unfortunately, not all domestic violence is reported to EMCC, this is an interesting result because most statistics used during lockdown to estimate the incidence of intimate partner violence were derived from Police reports and not all violent events reported to EMCC are reported to the police.

In order to produce results in a time frame compatible with the health emergency related to the recent lifting of lockdown measures, we used the samples from 2016 to 2018 for which a diagnosis was coded during the call by the medical assistant in charge of handling it. The ideal procedure would have been to perform a manual coding of this training sample, which was done for a sample of 39,907 reports from 2019, but retained in this work as a validation sample. Our previous work has shown that it takes about a thousand different examples to maximize the performance of the model [9]. This would have meant manually coding more than 100,000 notes, a task that was out of reach in a short period of time. The performances of the GPT-2 model measured with the manually coded validation samples were, however, very high and allowed us to derive a reason for call for all reports including the 22% of them with a missing value for the diagnosis.

Some limitations need to be acknowledged. First, although we have shown that an AI-based natural language model shows high performance in classifying free-text clinical reports, a small proportion of reports remains misclassified. Because our exercise here was to provide prevalence estimates, we adjusted the decision cutpoints so that precision equals recall. The bootstrap analysis showed that this was a very reliable strategy. Second, not all calls are handled by the EMCC, a proportion of them remains unanswered and this proportion increases during peak periods. It is therefore likely that around March 14 the number of attempted calls was higher than those handled. Finally, the study was done in Gironde, a department with a reportedly low rate of SARS-Cov-2 infection if compared to the Ile de France and the north-east regions of France. However, lockdown and fear of the epidemic affected all French people and the Gironde EMCC are the third largest in terms of the number of calls received in France, which has made it possible to build up a sufficiently large database.

## Conclusion

Major changes in the pattern of calls to EMCC were observed during the COVID-19 crisis in Gironde, starting for most of them a few days before the implementation of the lockdown and then gradually returning to pre-crisis levels around the date the containment was lifted. The observation of calls for flu-like symptoms anticipates an increase in ER admissions by about 14 days. The results of this study illustrate the extent to which automated classification of the reasons for calling EMCC is a powerful epidemiological surveillance tool, provides insights into the societal upheavals induced by a health crisis and would be instrumental to better anticipate the needs of the health care system.

## Supplementary Information

**Additional file 1:.** Example of real and synthetic EMS clinical reports.

## Data Availability

A subset of the dataset analysed during the current study can be made available from the corresponding author on reasonable request.

## Abbreviations

COVID-19: Coronavirus disease 2019
EHPAD: Etablissement d’hébergement pour personnes âgées dépendantes
EMCC: Emergency medical communication centers
ER: Emergency room
GPT-2: Generative pretrained transformer 2
INSERM: Institut national de la santé et de la recherche médicale
ISPED: Institut de santé publique et de développement
PCR: Reverse transcriptase-polymerase chain reaction
ROC: Receiver operating characteristic curve
SAMU: Service d’aide médicale urgente
SARS-CoV-2: Severe acute respiratory syndrome coronavirus 2
