# Supplementary material for: Trends in reasons for emergency calls during the COVID-19 crisis in the department of Gironde, France using artificial neural network for natural language classification
Source: Scand J Trauma Resusc Emerg Med. 2021 Mar 31;29:55. doi: 10.1186/s13049-021-00862-w (PMC8011068; doi:10.1186/s13049-021-00862-w)
Supplement: Supplementary file 1 — Additional file 1:. Example of real and synthetic EMS clinical reports. [file 13049_2021_862_MOESM1_ESM.docx]

**Supplementary material 1.** Example of real and synthetic EMS clinical reports.

| **Real EMS reports** |
| --- |
| homme de 68 ans douleurs abdominales dernière selle ce matin pas de gaz vomissement il y a une heure atcd subocclusions sur brides(2) atcd dnid/irénale  ap pour pole santé  pas d'ambulance de garde sur le secteur  tel ne repond pas  sp carence h 68 a  pas de pci  cst  fv 16  sat 94 aa  puls 83  ta 146/66 bd  vomissements |
| a raccroche avant conf gene respi je fais contre appel messagerie  je rappelle a nouveau je reussi a avoir la patiente  depuis vendredi grippe et gastro  vomissements ne s alimente pas  a fait malaise  pas de gene respi  vendredi médecin ttt / grippe et gastro vomissement ne s'alimente pas et ne boit pas non plus  vertige douluer épigastrique  >>>>sp en urgence  f76a dl ventre et estomac depuis 3 jrs vu med vendredi gastro dl 8/10 sat 95 cste fv26 ta g 124/65 pas de gene respi a pas manger depuis 3 jrs |
| a bu et elle vomit consciente  alcoolisée + toxique consciente >> ap pour hsa pas d'ap de garde sur secteur 8 sp carence f 16 a  alcolisee + drogue  puls70  ta 98/62 bd  sat 100 aa |
| ne mange pas / etourdi / diarrhée / s'urine dessus / ne veut pas aller a l'hopital diarrhée vertiges toux courbatures atcds : 0 ttt : 0 >> sos sos rappelle por demande de smur  cyanose  marbrures pas d'atcd appel pour asthénie / vertiges 8 de tension cyanosé marbré apyrétique point appel pulmonaire  mis sous 02 ==> mieux. on va envoyer sp  h 69 sos med sur place conscient orienté sos sur ll pupilles sym fv 26 diff sensation ettouff parole poss sat 84 cyanose marbrures fc115 reg ta g 80/50 mhta pas d'hospi pas de tt pas d'allergie 02 9l sat  sat a 93 refus des 4 pav et de libourne  demande exclusivement pellegrin regul r evrard hsa |
| lieu de travail pense fatigue malaise pci ne reagit pas mis en pls / insct respi /  f 34 a dit avoir etait droguer  cst puls 82  sat 100 aa  ta 127/87 |

| **Synthetic EMS reports** |
| --- |
| dl bras dt depuis dimanche / pas de fourmillement  F 41a  Douleur thoracique d'apparition brutale en se frottant les bras cette nuit sans faux mouvement  Bouge bien sa jambe ni l'estomac  Fumeuse  ATCD : néant  Douleur de novembre 2017 : RAS  Pas de dyspnée  pas de position antalgique  >> SPazl, doliprane 1000  f 41  f 41  dl bras dt  dl cou  cervicales  pas de céphalées  a deja eu ca mais pas consulté : peut etre pire  fc 57  ta 118/87  sat 99 |
| toux fievre  toux de nouveau  febrile 39  toux seche  pas de gene respi ni malaise  ATCD: neant  pas dautre symptome  vomissements x 1  >> conseils  >> rappel si besoin  rappel de la mere  toux  souhaite mdg dans un premier temps  virose selon la mere  rhino  toux rauque  lavage de nez  pas de gene respi  maman sur LL  toux rauque  pas d autres signes de gravite  mdg ok |
| chute / dl epaule dte / pas de pc / antcd prothese hanche dte  chute  a glissé  douleur épaule  adresser les sp  Bilan SP : h 90, dl epaule  conscient : oui  ATCD :  PLS : 72  TA : 132/67  Sat : 100  F Resp : 22 |
| sage femme du bras  elle dit qu'elle est triste  dit qu'elle ne peut pas se deplacer  f 50  cste  bien oriente  pas de pc  d apres sa femme de la dame a eu rale et fatigue  antcd pbl respi + stents en 2017  p 64  sat 100  ta 142/67  refuse evac  rappel sp : ne va pas bien dans cet etat  pas de plainte  est ok pour aller a l hopital |
| chute, plaie du front / assis par terre, assis sur le canapé, désorienté  chute  relevé par sa fille  a du mal a marcher  conscient  ****pompiers pour pellegrin  Bilan VSAV  H 96 ans  conscient  chute de sa hauteur  chariotale en bas  pas de douleur  a du mal a parler  bien oriente  FC 85  SAT 98  TA G 143/89  pas de trauma  pas de saignement  sous anticoagulant  pas de d'anticoagulant |
